# Hepatitis B virus X protein influences enrichment profiles of H3K9me3 on promoter regions in human hepatoma cell lines

**DOI:** 10.18632/oncotarget.12751

**Published:** 2016-10-19

**Authors:** Di-Yi Wang, Shu-Hong An, Lei Liu, Shan-Shan Bai, Kai-Xiang Wu, Rong Zhu, Zhao-Jin Wang

**Affiliations:** ^1^ Department of Pathology, Affiliated Hospital of Taishan Medical University, Taian 271000, China; ^2^ Department of Human Anatomy, Taishan Medical University, Taian, 271000, China; ^3^ Department of Pathology, The First people's Hospital of Taian, Taian, 271000, China; ^4^ Department of Pathology, Shanghai Medical College, Fudan University, Shanghai 200032, China

**Keywords:** hepatitis B virus X protein, histone H3 lysine 9 trimethylation, gene promoters, hepatocellular carcinoma

## Abstract

We previously showed that hepatitis B virus (HBV) X protein (HBx) could promote the trimethylation of histone H3 lysine 9 (H3K9me3) to repress tumor suppressor genes in hepatocellular carcinoma (HCC). In this work, we analyze 23,148 human promoters using ChIP-chip to determine the effects of HBx on H3K9me3 enrichments in hepatoma cells with transfection of HBx-expressing plasmid. Immunohistochemistry for HBx and H3K9me3 was performed in 21 cases of HBV-associated HCC tissues. We identified that H3K9me3 immunoreactivity was significantly correlated with HBx staining in HCC tissues. ChIP-chip data indicated that HBx remarkably altered promoter enrichments of H3K9me3 in hepatoma cells. We identified 25 gene promoters, whose H3K9me3 enrichments are significantly altered in hepatoma cells transfected HBx-expressing plasmid, including 19 gaining H3K9m3, and six losing this mark. Most of these genes have not been previously reported in HCC, and BTBD17, MIR6089, ZNF205-AS1 and ZP1 have not previously been linked to cancer; only two genes (DAB2IP and ZNF185) have been reported in HCC. Genomic analyses suggested that genes with the differential H3K9me3 enrichments function in diverse cellular pathways and many are involved in cancer development and progression.

## INTRODUCTION

Hepatitis B virus (HBV) chronic infection has been comfirmed as a major risk factor in the development of hepatocellular carcinoma (HCC) [1, 2]. Hepatitis B virus X protein (HBx), the product of the HBV gene HBX, is a pivotal factor in regulating gene transcription, participating in cell signaling, controlling cell proliferation, and immune response [2–4].

Histone modifications are essential and crucial for organizing the nuclear architecture, with subsequent effects on the regulation of gene transcription [5, 6]. Several studies have indicated that HBx is involved in the tumorigenesis of HCC via histone modifications [4, 6]. Histone H3 lysine 9 trimethylation (H3K9me3) is associated with gene silencing and serves as mechanism to inactivate tumor suppressor genes in various cancers [7–9]. Our previous studies demonstrated that HBx could promote both H3K9 trimethylation and DNA hypermethylation on the p16 promoter in hepatocarcinogenesis [10, 11]. These findings implicated that HBx-mediated H3K9me3 might be involved in the tumor suppressor gene silencing in carcinogenesis. However, the effects of HBx on H3K9me3 enrichment profiles on gene promoters were not fully understood.

Chromatin immunoprecipitation (ChIP) combined with genome tiling arrays (ChIP-chip) analysis is useful to determine epigenetic alterations in the genome [12, 13]. cDNA microarray has been extensively used as a convenient and cost-effective technique for analyzing gene expression signatures. In the present study, we adopted ChIP-chip technology to profile and compare variations of H3K9me3 enrichments on gene promoters in hepatoma cells with or without transfection of HBx-expressing plasmid to gain a better understanding of pathogenic mechanisms in HBV-associated HCC.

## RESULTS

### Expression of HBx and H3K9me3 in HBV-associated HCC tissues

**Figure 1:**
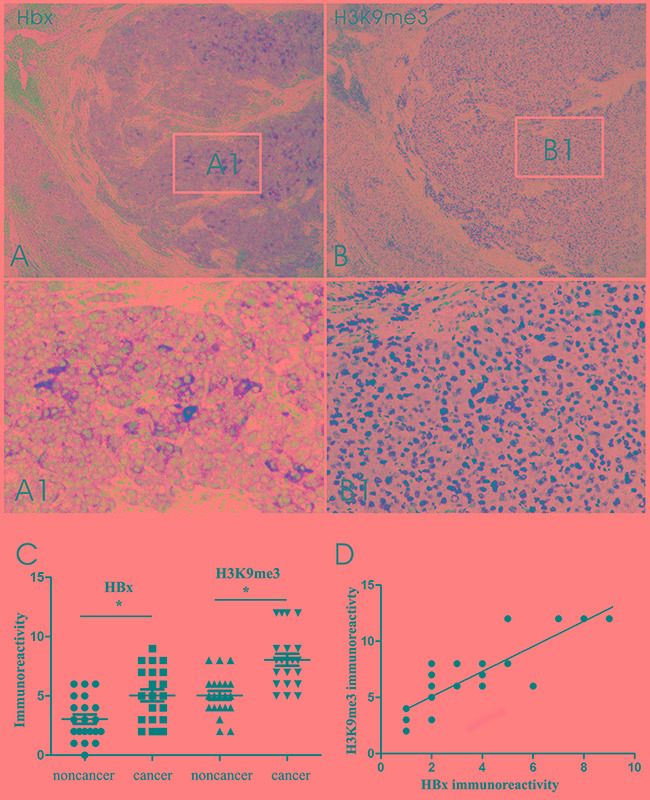
Representative HBx and H3K9me3 immunoreactivity in corresponding region of parallel sections from the same HCC case **A.** HBx immunoreactivity in HBV-associated HCC tissues and adjacent non-cancerous hepatic tissues. **A1.** Cytoplasmic HBx staining in the inset of A. **B.** H3K9me3 staining in the corresponding region of parallel section from the same HCC case. **B1.** Nuclear H3K9me3 staining in the inset of B (original magnifications ×40 [A and B], ×200 [A1 and B1]). **C.** Graph showing HBx and H3K9me3 immunoreactivity in HCC tissues and adjacent noncancerous hepatic tissues. Immunoreactivity was calculated by multiplying the percentage and intensity scores. Data indicate mean ± SEM. ^*^P < 0.05. **D.** Graph showing a positive correlation between HBx and H3K9me3 expression (Pearson analysis, r = 0.748, *P* < 0.05).

The expression of HBx and H3K9me3 in HBV-associated HCC tissue was determined by immunohistochemistry. HBx immunoreactivity displayed distinctive cytoplasmic staining (Figure 1A, A1) and H3K9me3 staining was observed strongly in the nucleus in HCC and adjacent non-cancerous tissues (Figure 1B, B1). Immunohistochemistry analysis showed that H3K9me3 expression and HBx staining in HCC were significantly higher than those in adjacent non-cancerous tissues (Figure 1C; *P* < 0.05). Pearson analysis showed that expression levels of H3K9me3 were significantly correlated with HBx staining in HBV-associated HCC tissues (Figure 1D, r = 0.748, *P* < 0.05).

### HBx increased the level of H3K9me3 in hepatoma cells

**Figure 2:**
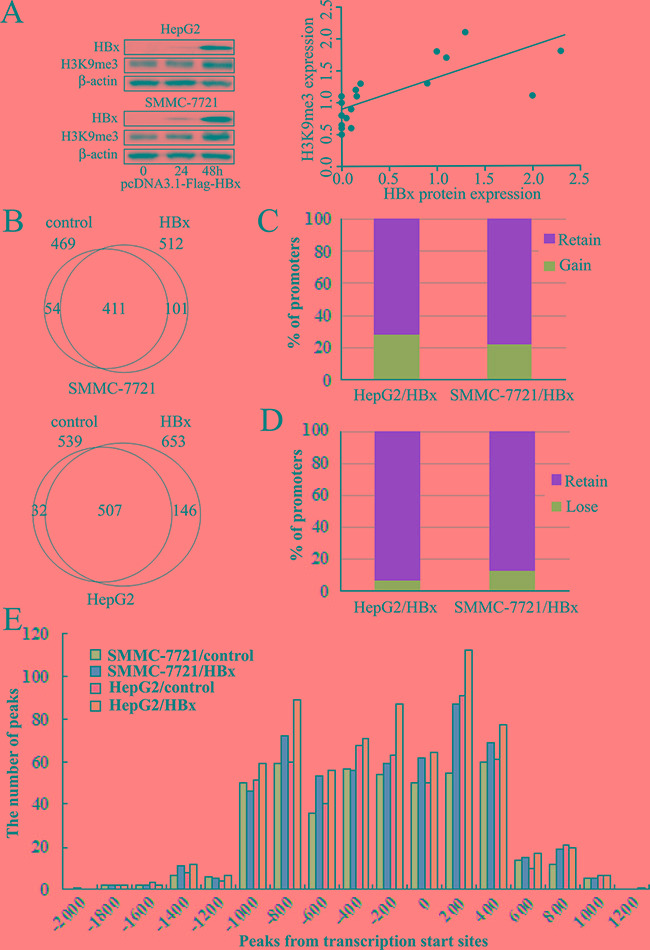
Alterations of H3K9me3 enriched promoters following HBx-expressing plasmid transfected into hepatoma cell lines for 48h **A.** Western blot analysis of HBx and H3K9me3 expression in SMMC-7721 and HepG2 cell lines with transfection of HBx-expressing plasmid. Representative histograms showing a positive correlation between HBx and H3K9me3 expression in hepatoma cells after transfection of HBx-expression plasmid (Pearson analysis, r = 0.791, *P* < 0.05). **B.** Venn diagraman analysis of gene promoters with enriched H3K9me3 in controls and HBx-expressing hepatoma cell lines. **C.** Percentage of genes with H3K9me3 that gains this mark “de novo” in HBx-expressing hepatoma cell lines. **D.** Percentages of genes that retain or lose H3K9me3 in HBx-expressing hepatoma cell lines, compared to controls. **E.** Distribution of H3K9me3 ChIP-chip peaks relative to gene TSSs. Shown are H3K9me3 peak frequencies relative to the distance from the nearest annotated TSS.

HepG2 and SMMC-7721 cell lines were transfected with HBx-expressing plasmid, and protein levels of HBx and H3K9me3 were detected by western blot analysis. No immunoblots for HBx was detected in both cell lines with control plasmid. The expression of HBx was detected at 24 h, significantly increased at 48 h in both cell lines after transfection of HBx-expressing plasmid (Figure 2A). We found that HBx could increase H3K9me3 levels in both cell lines, consistent with our previous study [11]. Pearson analysis showed that H3K9me3 levels were significantly correlated with HBx protein expression (Figure 2A, r = 0.791, *P* < 0.05).

### HBx influenced H3K9me3 enrichment profiles on promoters in hepatoma cells

To assess the enrichment pattern of HBx-mediated H3K9me3 on regulatory regions of genes, we used ChIP–chip to map profiles of H3K9me3 in HepG2 and SMMC-7721 cell lines after transfection of HBx-expressing plasmid for 48 h. Using a peak detection algorithm with a false discovery rate (FDR) of ≤ 0.05, enrichment profiles were compared to those of transfection with control plasmid. We identified a total of 653 and 512 H3K9me3-enriched promoters in HepG2 and SMMC-7721 after transfection of HBx-expressing plasmid, 539 and 469 in controls respectively (Figure 2B, Supplementary Table S1). HBx induced a gain of H3K9me3 on a substantial number of promoters in HBx-expressing hepatoma cells, but did not prevent loss of this mark from others. About 27.1% (146/539, HepG2) and 21.5% (101/469, SMMC-7721) of H3K9me3-marked genes gained this mark “de novo” when transfected with HBx-expressing plasmid (Figure 2B and 2C, Supplementary Table S1, S2 and S3). However, 5.9% (32/539, HepG2) and 11.5% (54/469, SMMC-7721) of H3K9me3-marked other genes lost this mark when transfected with HBx-expressing plasmid (Figure 2B and 2D, Supplementary Table S1, S2 and S3).

In addition, average H3K9me3 profiles are similar in controls and HBx-expressing hepatoma cells. Approximately 89% of H3K9me3 sites were mapped to proximal regions of transcription start sites (TSSs) of RefSeq genes (from about -1000 bp to +400 bp of TSSs), including many sites located in downstream proximal regions of TSSs in HBx-expressing hepatoma cells or controls (Figure 2E). However, there were substantial increases in the numbers of H3K9me3 enriched promoters in HBx-expressing hepatoma cells, compared to controls (Figure 2E, Supplementary Table S1).

### GO analysis of H3K9me3 enrichments induced by HBx

**Figure 3:**
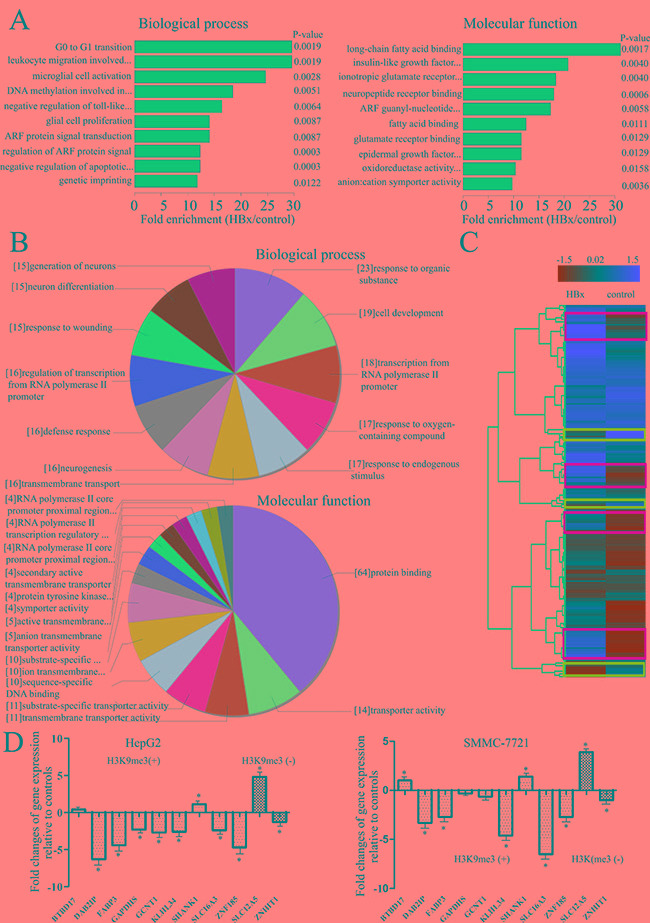
Enriched GO terms among genes which promoters were altered by HBx **A.** Shown are the Biological process or Molecular functional categories of enriched genes after transfection of HBx-expressing plasmid, compared with controls. Bar plots show the top ten fold enrichment values of the significant enrichment terms. **B.** Further analysis of genes enriched the GO terms linked to the Biological process or Molecular functional categories of enriched genes after transfection of HBx-expressing plasmid, compared with controls. Charts show the top ten counts of the significant enrichment terms. **C.** Representative example of Hierarchical cluster analysis of data between HBx-expressing hepatoma cells and empty-controls. Yellow squares indicate genes with a greater H3K9me3 enrichment in HBx expressing hepatoma cells relative to controls; blue squares indicate genes with a lower H3K9me3 enrichment in HBx-expressing hepatoma cells relative to controls. **D.** Quantitative RT–PCR analysis for differences in expression levels of H3K9me specific target genes between HBx-expressing hepatoma cells and controls in the subset of genes gaining or losing H3K9me3 on their promoters. Results were calculated by normalizing to GAPDH in the same sample with the ΔCt method. Changes in relative levels of gene mRNAs expressed as folds of controls. All values were mean ± SEM. ^*^P < 0.05 (n = 3).

To address the biological significance of enrichments in HBx-mediated H3K9me3 revealed by ChIP-chip, we identified Gene Ontology (GO) terms enriched among these genes. We found HBx-mediated enrichments in distinct terms for genes harboring this mark. Enriched GO terms for Biological process categories included G0 to G1 transition, leukocyte migration, DNA modification, and regulation of ARF protein signal transduction (Figure 3A; Supplementary Table S4). Enriched GO terms for Molecular function categories were mainly related to long-chain fatty acid binding, insulin-like growth factor (IGF) receptor binding, ARF guanyl-nucleotide exchange factor activity (Figure 3A; Supplementary Table S5).

Among 109 genes annotated with the enriched GO term ‘Biological process’, 19 have a role in cell development, 18 in regulation of transcription from RNA polymerase II promoter, and 16 in defense response (Figure 3B, Supplementary Table S4). Among 101 genes annotated with the GO enriched term ‘Molecular function’, 64 have a role in protein binding (including BAIAP3, DAB2IP, FABP3, KCNN1, KLHL34, MBD1, SHANK1, SLC12A5, and ZNHIT1), and 10 in sequence-specific DNA binding (Figure 3B, Supplementary Table S5). Most of genes are well-known oncogenes (e.g. BAIAP3, DAB2IP, and FABP3). Association with carcinogenesis-regulated genes seems therefore to be a feature of HBx-mediated H3K9me3 enrichment, however, we also found association of HBx-mediated H3K9me3 with metabolic processes (e.g. GAPDHS, GCNT1, and SLC16A3), which to our knowledge has not been reported earlier.

### HBx affected H3K9me3 enriched genes involved in tumorigenesis

To elucidate the role of putative H3K9me3 target genes in the tumorigenesis of HCC, we compared expression profiles of H3K9me3 enriched promoters that were differentially regulated in our experiments in controls and HBx-expressing cells. Genes enriching H3K9me3 mark were hierarchically clustered dependent on the gene H3K9me3 enrichment features of control against HBx-expressing cells (Figure 3C). The cluster of genes with higher H3K9me3 enrichments in HBx-expressing cells than in controls included DAB2IP, FABP3, SHANK1, and ZNF185. H3K9me3 losing genes included genes such as ZNHIT1 and SLC12A5. We identified nineteen H3K9me3 gaining genes and six H3K9me3 losing genes (Table 1) in both HepG2 and SMMC-7721 cell lines with transfection of HBx-expressing plasmid compared with controls. Most of these genes have not been previously reported in HCC, and BTBD17, MIR6089, ZNF205-AS1 and ZP1 have not previously been linked to cancer; only two genes (DAB2IP and ZNF185) have been reported in HCC. This clustering analysis of microarray data will indicate that the HBx-dependent transcriptional regulation is closely associated with the development of HCC.

**Table 1:** HBx induced H3K9me3 enrich promoters in hepatoma cells identified by CHIP-chip

Gene ID	Gene name	Peak region	Peak To TSS	Fold enrichment (vs. control)	P-value
**Binding sites gain of H3K9me3**					
NM_177947	ARMCX3	chrX: 100878290-100878762	76	2.55	0.0352
NM_001080466	BTBD17	chr17: 72357893-72359054	-515	5.83	0.0414
NR_033380	CD99P1	chrX: 2526402-2527078	-426	2.01	0.0207
NM_032552	DAB2IP	chr9: 124328114-124329861	-392	15.56	0.0003
NM_024045	DDX50	chr10: 70660864-70661331	64	3.65	0.0345
NM_004102	FABP3	chr1: 31845858-31846522	-267	16.11	0.0028
NM_014364	GAPDHS	chr19: 36024175-36024695	122	5.82	0.0366
NM_001097634	GCNT1	chr9:79056359-79056928	62	7.78	0.0028
NM_153270	KLHL34	chrX: 21676377-21677101	-291	7.23	0.0350
NM_001204143	MBD1	chr18: 47808786-47809249	-880	2.07	0.0352
NR_106737	MIR6089	chrX: 2526402-2527355	-352	2.23	0.0207
NM_001144856	PLEKHG6	chr12: 6419164-6419624	-207	2.55	0.0200
NM_031915	SETDB2	chr13: 50018436-50019317	448	2.04	0.0495
NM_016148	SHANK1	chr19: 51219710-51220370	155	10.31	0.0055
NM_001206951	SLC16A3	chr17: 80185203-80185940	-93	4.82	0.0095
NM_032701	SUV420H2	chr19: 55850507-55851187	-373	2.26	0.0488
NM_001178113	ZNF185	chrX: 152086241-152086768	96	5.32	0.0273
NR_119385	ZNF295-AS1	chr21: 43441038-43441929	-628	2.66	0.0287
NM_207341	ZP1	chr11 60634137- 60635106	-392	2.29	0.0371
**Binding sites loss of H3K9me3**					
NM_001031722	ATAT1	chr6: 30593317- 30593582	-1164	2.24	0.0096
NM_001199096	BAIAP3	chr16: 1386416- 1386676	349	2.02	0.0232
NM_002248	KCNN1	chr19: 18060811- 18061289	-1060	3.20	0.0076
NM_020708	SLC12A5	chr20: 44657861- 44658166	193	9.72	0.0076
NM_006062	SMYD5	chr2: 73440336- 73440814	-790	3.03	0.0145
NM_006349	ZNHIT1	chr7: 100859752-100860221	-997	14.29	0.0019

### HBx altered the gene expression in hepatoma cells

We next addressed the impact of gain or loss of H3K9me3 induced by HBx on gene expression. We selected a subset of genes annotated with the GO terms enriched among genes gaining or losing H3K9me3 (at least 4-fold change), and assessed their expression at 48 h after transfection of HBx-expressing plasmid by qRT-PCR. Results showed that most of genes gaining H3K9me3 became significantly downregulated, and genes losing this mark were significantly upregulated compared to controls (Figure 3D). However, altered H3K9me3 on promoters did not have a pronounced effect on some gene expression.

## DISCUSSION

In the present study, we used a ChIP-chip microarray, which enables the genome-wide identification of binding sites of transcription factors and chromatin regulators [12, 13], and examined the influence of HBx on H3K9me3 enrichment profiles on promoter regions to test the hypothesis that H3K9me3 was associated with the pathogenesis of HBV-associated HCC [10, 11]. We found that H3K9me3 immunoreactivity levels were remarkably correlated to those of HBx in HCC tissues, and HBx was able to upregulate H3K9me3 levels in hepatoma cell lines. CHIP-chip data showed that HBx could substantially increase the number of H3K9me3-encriched promoters but did not prevent this mark loss on others, suggesting that HBx could alteration enrichment profiles of H3K9me3 at promoters in a gene-specific manner.

In order to obtain insights into H3K9me3 target gene function, GO analysis annotation were applied to the H3K9me3 target gene pool. GO analysis showed H3K9me3 enrichments on gene promoters were significantly influenced by transfection of HBx-expressing plasmid. GO terms for Biological process categories included G0 to G1 transition, leukocyte migration, DNA modification, and regulation of ARF protein signal transduction. Most of genes are well-known oncogenes (e.g. BAIAP3, DAB2IP, FABP3, and ZNF185) [14–16]. On the other hand, GO terms for Molecular function categories were mainly related to long-chain fatty acid binding, IGF receptor binding, ARF guanyl-nucleotide exchange factor activity. Previous report showed that HBx could promote hepatic steatosis through up-expressing liver fatty acid binding protein [17]. However, fatty acid-binding protein was down-expressed in HCC and correlated with tumor differentiation and metastases [18]. The IGF system is involved in regulation of proliferation, differentiation, and apoptosis in the development of cancers [19]. ARF guanyl-nucleotide exchange factor plays a major role in mediating receptor endocytosis, Wnt signaling, and tumor metastases [20]. Therefore, alterations of this repressive mark induced by HBx could have severe consequences in hepatoma cell biology.

Among identified candidate genes, DAB2IP is a Ras GTPase-activating protein and plays a cancer suppressor role in tumorigenesis. The expression of DAB2IP is obviously low in HCC patients and inhibition of DAB2IP could contribute to HCC cell proliferation and invasion in vitro [14]. FABP3 may be involved in fatty acid transport, cellular differentiation and proliferation, signaling transduction, and gene transcription. Up-regulation of FABP3 could result in mitochondrial dysfunction and lead to apoptosis [15]. ZNF185 may function as a tumor-suppressor protein by associating with the actin-cytoskeleton [16]. Study confirmed the down-regulation of ZNF185 gene in the drug-tolerant HCC cells with IFN-alpha treatment [21]. ZNHIT1 plays an important role in facilitating transcriptional activation during cell differentiation by a p38 MAPK-mediated signal mechanism [22]. In the present study, we found that H3K9me3 enrichments on promoters of DAB2IP, FABP3, ZNF185 and ZNHIT1 displayed obvious alteration in HBx-expressing hepatoma cells, which would provide a better understanding of cell differentiation and proliferation abnormalities involved in the HBV-associated HCC patients.

SHANK1 may play tumor-associated roles in the development of malignant neoplasm [23]. The function of the sharpin (shank-associated RH domain interacting protein) may be involved in regulation of small GTPase-mediated signaling pathways that may be required for NLRP3 (NLR family, pyrin domain containing 3) inflammasome activation and possibly plays active roles in immune cell proliferation and differentiation [24, 25]. Previous report showed that SLC16A3 might be involved in the tissue-specific transplantation antigen P35B (TSTA3)-activated network mediated immune response in hepatitis/cirrhotic tissues (HBV or HCV infection), such as T-cell homeostasis, neutrophil chemotaxis, interleukin-8 expression, immune response, and B-cell activation [26, 27]. Our results showed that HBx could increase H3K9me3 enrichment on genes of SHANK1 and SLC16A3, this might contribute to modulation of tumor-immune cell interactions.

Association with carcinogenesis-regulated genes seems therefore to be a feature of HBx-mediated H3K9me3 enrichments, however, we also found association of H3K9me3 with metabolic processes, such as genes GAPDHS, GCNT1 and SLC16A3, which to our knowledge has not been reported earlier in HBV-associated HCC. Study has found that GAPDH itself or GAPDH-related proteins may be involved in lung tumorigenesis [28]. The expression of GCNT1 has strong correlation with tumorigenicity, development and metastasis in human prostate cancers [29, 30]. Furthermore, recent study has identified that DNA methylation of the SLC16A3 promoter is involved in the regulation of monocarboxylate transporter 4 (MCT4) in renal cancer resulting in cell apoptosis [31]. In this study, we have demonstrated that these H3K9me3 enriched genes are not association with metabolic processes, but rather that they possibly play active roles in tumorigenesis. Since these gene expressions were most affected in cell proliferation and differentiation, signal transduction and transcription, it will be interesting to elucidate the effect of these genes on HBV-associated HCC tumorigenesis in future studies.

LncRNAs and miRNAs play a crucial role in the regulation of gene expression and modulation of signaling pathways, which contribute to the pathogenesis and progression of HCC [32]. Our results showed that HBx-mediated H3K9me3 enrichments were obviously up-regulated on promoters of lncRNAs CD99P1, ZNF295-AS1 and miRNA MIR6089. Previous study demonstrated that silencing CD99P1 inhibited proliferation and differentiation of lung fibroblasts [33]. ZNF205-AS1 and MIR6089, to our knowledge, have not been reported earlier in cancers.

In summary, we systematically evaluated HBx-mediated H3K9me3 enrichments in hepatoma cells with transfection of HBx-expressing plasmid and gained new insights into links between key genes and histone methylation induced by HBx in the development of HCC. Genomic analyses suggested that genes with the differential H3K9me3 enrichments induced by HBx function in diverse cellular pathways and many are involved in cancer development and progression. These novel candidate genes showed here may become potential biomarkers or future therapeutic targets. Further investigations are needed to clarify roles of identified H3K9me3 candidate genes in the pathogenesis of HBV-associated HCC.

## MATERIALS AND METHODS

### Cell culture and transfection

Human heptoma cell lines (SMMC-7721 and HepG2) were purchased from the Cell Bank of the Chinese Academy of Sciences (Shanghai, China). HBx-expressing plasmid (pcDNA3.1-Flag-HBx) and control plasmid (pcDNA3.1) sequences, and transient transfection have been described previously [10, 11, 34]. Every cell line was transfected with 0.5 μg/well of HBx-expressing plasmid or control plasmid for 48h in 96-well plates. HBx-transfected and empty-plasmid control cells are kept under identical culture conditions, expression analyses are performed at same passage numbers.

### Protein extraction and western blot analysis

Cells were lysed, and protein was extracted as previously described [10, 11]. Protein lysate from each sample was separated electrophoretically in sodium dodecyl sulfatepolyacrylamide gel, and then transferred to polyvinylidene fluoride (PVDF) membranes. Western blot analyses were performed with anti-HBx and anti- H3K9me3 (Abcam, Cambridge, UK).

### Chromatin immunoprecipitation (ChIP), DNA labelling and array hybridization

HepG2 and SMMC-7721 cell lines transfected with HBx-expressing plasmid (2 biological replicates per cell lines) were chemically crosslinked, resuspended in lysis buffer, and sonicated fragments ranged in size from 200 to 1000 bp (Supplementary Figure S1A). Sonicated chromatin was resuspended in IP buffer and incubated with magnetic beads conjugated H3K9me3 antibody (Abcam, Cambridge, UK). An un-enriched sample of DNA was treated in a similar manner to serve as input. The immunoprecipitated DNA was tested for enrichment of control loci by qPCR validation (primers and results are listed in Supplementary Table S6 and Supplementary Figure S1B, respectively). IgG control levels were below the threshold. The puriﬁed ChIP and input DNA was amplified and then fluorescently labeled using the NimbleGen Dual-Color DNA Labeling Kit, which is a single array designs that includes 23,148 gene promoter regions (from about -1300 bp to +500 bp of TSSs) totally covered by ~180,000 probes with approximately 210 bp spacing, dependent on the sequence composition of the region. The Cy5-ChIP and Cy3-input labelled DNA samples were co-hybridized to ArrayStar Human RefSeq Promoter Arrays, post hybridization washes were carried out and microarrays were scanned using Agilent Scanner G2505C.

### ChIP-chip data analysis

Signal intensity data were extracted from scanned images using the NimbleScan v2.5 (Roche-NimbleGen). Log2 ChIP/Input ratios were scaled and centered around zero by subtracting the bi-weight mean for the log2 ratio values for all features on the array from each log2 ratio value. NimbleScan detects peaks by searching for 4 or more probes whose signals were above the specified cutoff values, ranging from 90% to 15%, using a 1,000 bp sliding window. The cutoff values are a percentage of a hypothetical maximum, which is the mean + 6 [standard deviation]. The ratio data is then randomized 20 times to evaluate the probability of “false positives”. Each peak is then assigned a FDR score based on the randomization. The MA plots and box-plots were applied to assess the quality of raw data and effect of normalization (Supplementary Figure S1C and S1D). A correlation matrix was used to describe correlation between replicate experiments (Supplementary Figure S1E).

### Hierarchical cluster analysis for H3K9me3 enrichment promoters

Hierarchical cluster analysis was performed for **e**nriched genes selected by our experiments. Log2-ratio data for both genes gain and loss H3K9me3 were clustered in a manner dependent on the gene H3K9me3 enrichment features of HBx-expressing hepatoma cells and empty-controls, using a hierarchical clustering by Cluster 3.0 software [35].

### Gene ontology analysis

Gene ontology (GO) term enrichments within a target gene set were calculated using Gene Ontology (www.geneontology.org), which provides three structured networks of defined terms that describe gene product attributes. The *P*-value denotes the significance of GO Term enrichment in the differentially expressed gene list (*P* < 0.05 was considered statistically significant).

### Quantitative reverse transcription–PCR (qRT–PCR) analysis

The expression profiles of genes selected from enriched GO terms that showed the largest differences (Table 1) were assessed by qRT-PCR at 48 h after transfection of HBx-expressing plasmid. Total RNA was isolated from cells using TRIzol reagent (Invitrogen) according to the manufacturer's protocol. cDNA was synthesized using a cDNA synthesis kit (Invitrogen) according to the manufacturer's instructions. Primer sequences are listed in the Supplementary Table S6. qRT–PCR was performed in triplicates by using a 7300 real-time PCR system (Applied Biosystems, Foster City, CA) according to the manufacturer's instructions.

### Immunohistochemistry analysis

Immunohistochemical analyses were performed essentially as described before [10, 11]. Briefly, twenty-one HBV positive HCC specimens were fixed in 10% buffered formalin. Parallel paraffin sections were stained with anti-HBx and anti-H3K9me3 (Abcam, Cambridge, UK), and then counterstained with hematoxylin. Immunoreaction scores were calculated by multiplying the percentage and intensity scores as our previously described [11].

### Statistical methods

All results are expressed as mean ± SEM. Statistical analysis was performed for the comparison of two groups in the microarray, immunoreactivity, and analysis of variance for comparisons was performed using the Student's t-test. Differences with *P* < 0.05 were considered statistically significant. Correlation between HBx and H3K9me3 was analyzed using Pearson rank correlation.

## SUPPLEMENTARY MATERIALS FIGURES AND TABLES












