# Supplementary material for: Hepatitis B virus X protein influences enrichment profiles of H3K9me3 on promoter regions in human hepatoma cell lines
Source: Oncotarget. 2016 Oct 19;7(51):84883–92. doi: 10.18632/oncotarget.12751 (PMC5356706; doi:10.18632/oncotarget.12751)
Supplement: Supplementary file 1 [file oncotarget-07-84883-s001.pdf]

Hepatitis B virus X protein influences enrichment profiles of H3K9me3 on promoter regions in human hepatoma cell lines

SUPPLEMENTARY FIGURE AND TABLES

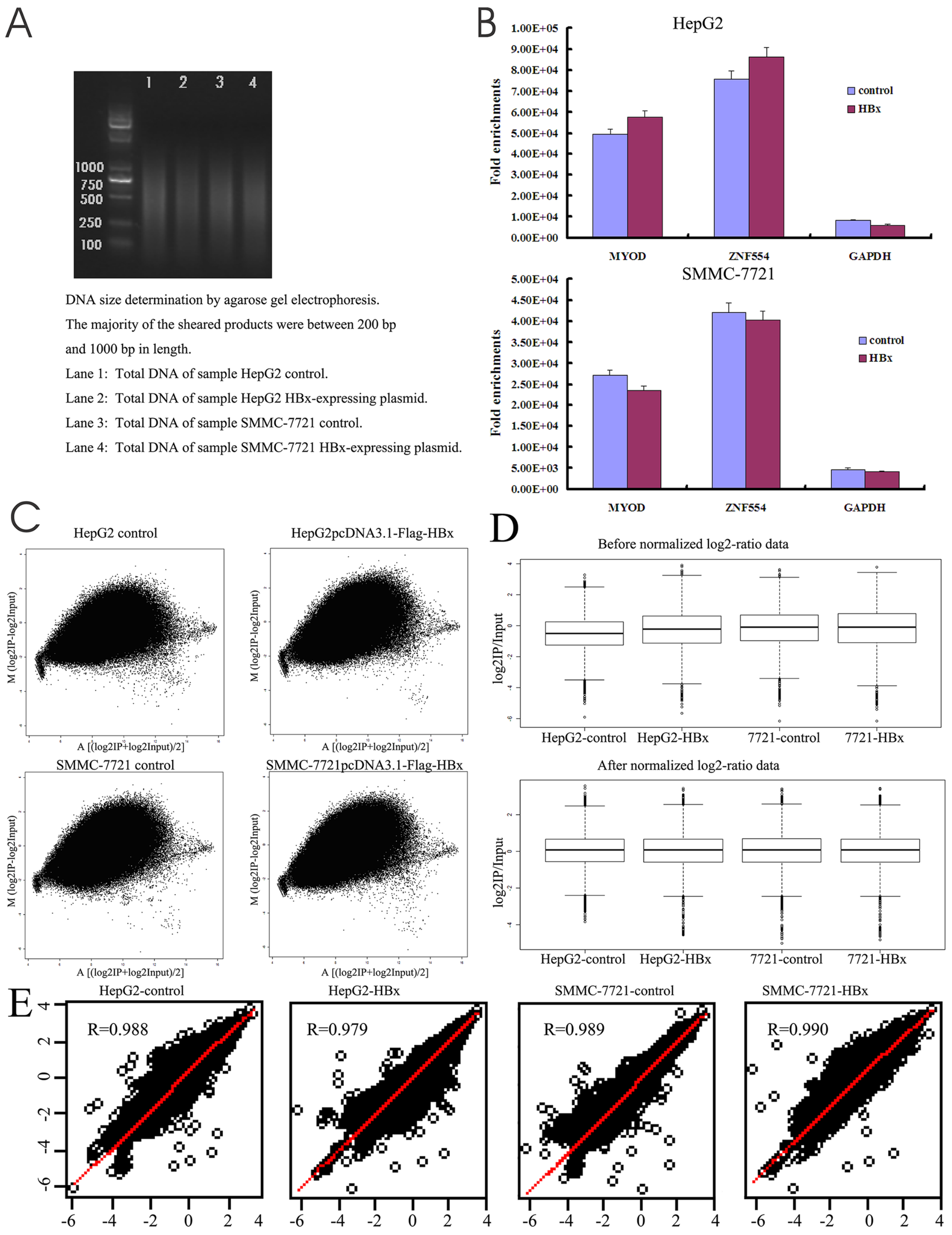

Supplementary Figure S1: Reproducibility of H3K9me3 ChIP-chip.

Supplementary Table S1: Data analysis of results.

See Supplementary Table S1

Supplementary Table S2: Data analysis of results - The number of peaks overlapping the promoter region for the corresponding sample.

See Supplementary Table S2

Supplementary Table S3: Data analysis of results - The number of peaks overlapping the promoter region for the corresponding sample.

See Supplementary Table S3

Supplementary Table S4: Biological process results.

See Supplementary Table S4

Supplementary Table S5: Molecular function results.

See Supplementary Table S5

Supplementary Table S6: The primers used in quantitative RT-PCR

Primers	Forward	Reverse	Amplicon size (bp)
BTBD17	5′-GCTGGCACCTCCATCAACC-3′	5′-GCTCCCCGCAGTACAGGTA-3′	268
DAB2IP	5′-GTACCGGGAGACCGACAAGA-3′	5′-GATGCGGATCATGGGTCCAG-3′	166
FABP3	5′-CATGACCAAGCCTACCACAAT-3′	5′-CCCCAACTTAAAGCTGATCTCTG-3′	100
GAPDHS	5′-TGTGGGCATCAATGGATTTGG-3′	5′-ACACCATGTATTCCGGGTCAAT-3′	116
GCNT1	5′-AGCGGTATGAGGTCGTTAATGG-3′	5′-GACCACGAAGTAGGCACTGC-3′	104
KLHL34	5′-CACTGTAGAGGACACTCTGGA-3′	5′-CGAAGCAGCAGTTCTCTGGA-3′	110
SHANK1	5′-AGTTCCGATACAAGACCCGAG-3′	5′-CCGAGCTGCACATACTCCA-3′	112
SLC16A3	5′-CGGCTTTGTGCTTTACGCC-3′	5′-GCTGAAGAGGTAGACGGAGTA-3′	220
ZNF185	5′-AGCTCTACCACCAAAGGGATT-3′	5′-TGGCGAATGAGTCCTCAATGC-3′	121
SLC12A5	5′-GCACCATCGAAATCCTGCTG-3′	5′-GTGCCGTAAACACGCATGT-3′	112
ZNHIT1	5′-CTCAGCTCGGCAAGAGACTG-3′	5′-GCCGTCAGGTAGTTAGGGC-3′	166
**ChIP-PCR**			
GAPDH	5′-CCACAGTCCAGTCCTGGGAACC-3′	5′-GAGCTACGTGCGCCCGTAAAA-3′	183
MYOD	5′-GCCACAACGGACGACTTCT-3′	5′-GAGTGCTCTTCGGGTTTCAG-3′	119
ZNF554	5′-CGGGGAAAAGCCCTATAAAT-3′	5′-TCCACATTCACTGCATTCGT-3′	118
